# Practical Identifiability in a Viscoelastic Respiratory Model for Mechanical Ventilation

**DOI:** 10.1007/s11538-025-01497-z

**Published:** 2025-08-06

**Authors:** A. E. Cerdeira, N. N. Lam, S. Hamis, P. D. Docherty

**Affiliations:** 1 Department of Mechanical Engineering, University of Canterbury, Christchurch, New Zealand; 2 Division of Systems and Control, Department of Information Technology, Uppsala University, Uppsala, Sweden

**Keywords:** Practical identifiability, Identifiability, Respiratory mechanics, Profile likelihood

## Abstract

Mechanical ventilation is a life support system for patients with acute respiratory distress syndrome (ARDS). As part of strategies to protect the lung during ventilation, plateau pressure can be determined via an end-inspiratory pause; however, there is no agreed-upon pause duration in medical protocols. Mechanical ventilation can be modelled using the Viscoelastic model (VEM) for respiration. The identification of static compliance is of clinical interest, as it can be used to estimate plateau pressure. Practical identifiability analysis quantifies the confidence with which model parameters can be estimated from finite, noisy data. This paper evaluates the robustness of plateau pressure estimates in clinical data by analysing practical identifiability of the VEM identified in data with varying durations of end expiratory pauses. Profile likelihood and Hamiltonian Monte Carlo (HMC) simulations were used to determine estimation robustness. The methods were applied to mechanical ventilation data from a previous ARDS study. Profile likelihood and HMC showed strong agreement in both parameter estimates and identifiability results with similar confidence distributions. Both methods demonstrated a loss of parameter robustness that would preclude clinical utility when the end expiratory pause was reduced. By quantifying the confidence in parameter estimation and finding trade-offs in parameters that may be previously unknown when parameters are estimated, the methods give insight into the certainty of the estimate and parameter behaviours, even when the model fits the data well.

## Introduction

Mechanical ventilation in the intensive care unit (ICU) for patients experiencing acute respiratory distress syndrome (ARDS) provides gas exchange while resting respiratory muscles (Silva et al. 2023). However, suboptimal ventilator settings can lead to ventilator induced lung injury (VILI) (Slutsky and Ranieri 2013; Pelosi et al. 2021; Ang et al. 2022; Beitler 2019; Amato et al. 1998). Plateau pressure (*P*_*plat*_), is the pressure in the alveoli at the end of inspiration. It cannot be measured in real time at the bedside but can be either calculated, as the inspired volume divided by the lung static compliance, or estimated using ventilator settings (Buckley et al. 2007; Mora Carpio and Mora 2024). *P*_*plat*_ is a clinically significant value as it is a key contributor to barotrauma and volutrauma, both which can lead to induced sterile inflammation and multiorgan failure (Beitler 2019) if not appropriately managed. Thus, monitoring changes in plateau pressure can also help determine lung condition over time. For patients with ARDS, mechanical ventilation strategies target a *P*_*plat*_ below 30 cmH_2_O to avoid VILI (Pelosi et al. 2021; Beitler 2019).

To estimate plateau pressure, the patient’s lungs are inflated, then the expiratory port is closed and flow ceased to create an end-inspiratory pause (EIP) (Slutsky and Ranieri 2013). Currently, there is no agreed-upon length to this pause, and variations from 0.5 s to 30 s are observed over varying medical protocols and different ventilator types (Barberis et al. 2003; Hess and Tabor 1993). Extending this pause can reduce gas exchange (Battistella et al. 1985) and thus clinicians have sought to decrease the pause duration.

Multiple strategies exist to model respiration physiologically under mechanical ventilation using lumped parameter models (Bates 2009; Laufer et al. 2017; Morton et al. 2019). Parameters of interest that determine the transfer of mechanical energy to the lung are airway resistance and lung tissue elastance (or inversely, compliance) (Ang et al. 2022; Bates 2009; Laufer et al. 2017; Schranz et al. 2012). First order models (FOM) use airway resistance, air flowrate into the lungs, and a constant airway and lung tissue elastance to capture respiration mechanics (Ang et al. 2022; Laufer et al. 2017; Sun et al. 2021; Langdon et al. 2016). More complex models add a time-varying lung elastance to track dynamic tissue variation throughout the breath (Ang et al. 2022; Morton et al. 2019). Alternatively, the behaviour missed by the FOM can also be modelled through static and viscoelastic terms using the two-compartment viscoelastic model (VEM) (Schranz et al. 2012).

The VEM captures lung static and viscoelastic resistance and compliance using four parameters which can be estimated from measured airway pressure data. However, there is limited information on whether the estimated parameters are reliable when identified from typical noisy clinical data. Parameter trade-offs can occur, where the change in modelled pressure caused by manually perturbing one parameter can lead to very similar modelled behaviour with concomitant, specific changes in other parameters (Lam et al. 2022). To address the potential for trade-offs, an identifiability analysis in tandem with parameter identification can quantify confidence in the estimated parameters.

Limitations in the quantity and quality of data can impact practical model identifiability (Lam et al. 2022, 2024; Wieland et al. 2021; Villaverde 2025). Practical identifiability concepts and approaches can be used as a foundation to develop data collection protocols to improve parameter inference. Commonly used methods include profile likelihoods (PL) and Monte Carlo simulations (Lam et al. 2022). Profile likelihood explores the parameter space for each parameter in the direction of least error increase for each individual parameter (Raue et al. 2009). The approach develops confidence intervals that indicate non-identifiability if the interval extends infinitely. Another practical identifiability method is the Hamiltonian Monte Carlo (HMC) method, a type of Markov Chain Monte Carlo sampling method that generates sequential random samples to generate posterior probability density distributions of the parameters (Ravenzwaaij et al. 2016; Stan Development Team 2025). Previous studies have shown that PL is faster, but HMC can provide more information on parameter behaviour within the model (Simpson et al. 2020).

This paper uses profile likelihood and HMC to determine how practical identifiability changes as the EIP duration is reduced in a viscoelastic respiratory model. The methods are applied to mechanical ventilation data from a previous ARDS study (Stahl et al. 2006). The identification of static compliance is of clinical interest, as it is used in the estimation of *P*_*plat*_, with focus given to the analysis of the identifiability of the VEM and thus confidence of estimated parameters for clinical applications. The study aims to understand how identifiability varies across the cohort, quantify how EIP reduction impacts identifiability, particularly the conditions required to find an accurate estimate of *C*_1_ and therefore *P*_*plat*_, and compare PL and HMC outcomes for practical identifiability analysis. Success will be demonstrated by achieving comparable parameter estimates between PL and HMC, and by observing similar trends in parameter identifiability loss across simulated and patient data for both PL and HMC. Beyond these metrics, the findings are expected to provide an understanding of practical identifiability of the VEM and apply current practical identifiability methods to a physiological model.

## Methods

**Fig. 1:**
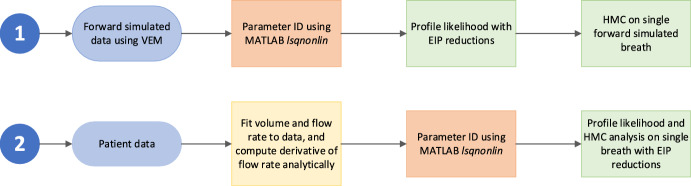
Flow chart of paper methodology

Figure 1 shows a flow chart of the methodology used in this paper.

Initially, mechanical ventilation pressure and volume data was forward simulated using the VEM. The simulated timespan for the in silico data was then truncated to observe the effect of reducing EIP on parameter identifiability. Profile likelihood analysis was then conducted on the truncated simulated data to observe changes in identifiability.

Subsequently, parameter estimation, using MATLAB’s nonlinear fitting solver (The MathWorks Inc 2024), was carried out for mechanical ventilator breath data with extended EIPs to characterise the parameter values over the population. Then, an exemplar breath among the cohort was identified and HMC simulation methods in RStan were performed to determine the degree of agreement between HMC and profile likelihood methods (Stan Development Team 2024; Stan Development Team 2024). This exemplar breath (from patient data) was then truncated to reduce EIP and observe changes in identifiability in clinical data.

### Data

19 datasets from mechanically ventilated patients in a previous ARDS study were used for validation (Stahl et al. 2006). The study was approved by local ethics committees of the participating universities and informed consent was obtained from each patient or their legal representative (Schranz et al. 2012; Stahl et al. 2006). Each dataset consisted of flow rate and airway pressure signals sampled at 125 Hz for approximately 3–4 h. The data contained indices for the inspiratory and expiratory portion of individual breaths. Since all patients were sedated, breathing mechanics were controlled by the ventilator and the indices could be relied upon to define the duration of breaths. One breath was defined as the period from the start of inspiration to the end of expiration, but this study isolated and only used data from the inspiratory phase of the breath.

The dataset was intended to exhibit respiratory mechanics across a broad range of different ventilator settings. Hence, not all breaths contained EIPs, and those that did had variable durations of EIP. Consequently, breaths were only selected for analysis if they were over 4 s in duration and had peak inspiratory values between 10 and 40 cmH_2_O. 73 breaths, from 12 of the 19 patients, matched these selection criteria. From the 73 cohort breaths, a breath where parameters were highly identifiable was further selected as an exemplar for HMC analysis and comparison of the two methods.

### Forward Simulation

**Fig. 2:**
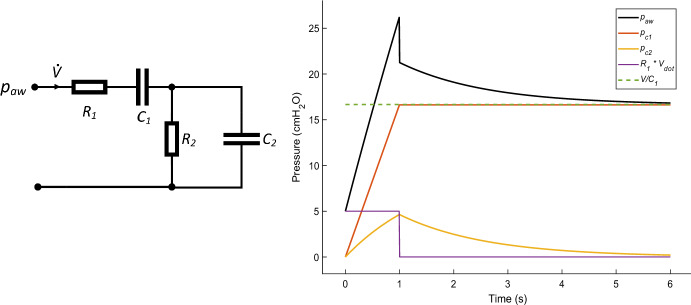
(Left) an analogous circuit diagram of the viscoelastic model is show, where *R*_1_ and *C*_1_ are the static resistances and compliance, while *R*_2_ and *C*_2_ are the viscoelastic components. $\dot{{{V}}}$ is the model input and *p*_*aw*_ the model output. (Right) simulated lung pressure in response to 500 mL/s flow input, with one second inspiratory phase and 5 s EIP. Model components are shown. Airway pressure can be seen to decay to a plateau pressure corresponding to approximately *V/C*_1_. Reduction in end-inspiratory pause reduces the available data for parameter identification

A priori structural identifiability, a prerequisite for parameter estimation and practical identifiability, is known for the Viscoelastic Model (Schranz et al. 2012; Lam et al. 2022; Villaverde et al. 2016). The model has four patient specific parameters, ***θ*** = (*R*_1_*, C*_1_*, R*_2_*, C*_2_). The VEM is shown in Eqs. (1) and (2) as

1 $$\begin{array}{c}\left[\begin{array}{c}{\dot{p}}_{c1}\\ {\dot{p}}_{c2}\end{array}\right]=\left[\begin{array}{cc}0& 0\\ 0& -\frac{1}{{R}_{2}{C}_{2}}\end{array} \right] \left[\begin{array}{c}p{}_{c1}\\ {p}_{c2}\end{array}\right]+\left[\begin{array}{c}\frac{1}{{C}_{1}}\\ \frac{1}{{C}_{2}}\end{array}\right]\dot{V}\end{array}$$

2 $$\begin{array}{c}{p}_{aw}={p}_{c1}+{p}_{c2}+{R}_{1}\dot{V} \end{array}$$

where: *p*_*c*1_ is the alveolae pressure [cmH_2_O];* p*_*c*2_ is the upper lung pressure [cmH_2_O]; and parameter *R*_1_ is the static resistance [cmH_2_O·s/mL]; *C*_1_ is the static compliance [mL/cmH_2_O]; *R*_2_ is the viscoelastic resistance [cmH_2_O·s/mL]; *C*_2_ is the viscoelastic compliance [mL/cmH_2_O]; *p*_*aw*_ is the measured airway pressure [cmH_2_O]; $\dot{V}$ is the flow rate [mL/s]. Figure 2 shows a breath forward simulated over 6 s and the analogous electrical circuit of the model.

For the in silico data, the VEM was simulated by applying a constant flow rate, $\dot{V}=$ 500 mL/s, over 1 s to simulate inflation of the lungs, then applying a 4s EIP. A physiologically feasible parameter set of ***θ*** = (0.010, 30.00, 0.020, 80.00) was selected for the initial estimate (Schranz et al. 2012). *P*_*plat*_ can be approximated by *V/C*_1_, while *p*_*C*2_(0) was found to be the initial pressure value, corresponding to the Positive-End-Expiratory-Pressure (PEEP). Equation 1 was solved analytically, with solutions shown in (3) and (4):

3 $$\begin{array}{c}{p}_{c1}\left(t\right)=\frac{V\left(t\right)-V\left(0\right)}{{C}_{1}}+{P}_{c1}\left(0\right),\end{array}$$

4 $$\begin{array}{c}{p}_{c2}\left(t\right)={e}^{-\frac{t}{{R}_{2}{C}_{2}}} \left[{p}_{c2}\left(0\right)+ {\int }_{0}^{t}{e}^{\frac{t}{{R}_{2}{C}_{2}}}\left(\frac{\dot{V}}{{C}_{2}}\right) dt\right].\end{array}$$

### Parameter Estimation

The MATLAB non-linear least squares solver *lsqnonlin* (LSQNL) was used for parameter identification. The default settings of ‘StepTolerance’ = 1e−6, ‘OptimalityTolerance’ = 1e−6 were used. All other settings were left as default. Parameter identification on the 73 patient breaths was performed to characterize the patient parameters across the dataset. Parameter supports can be found in Appendix A.

### Practical Identifiability

For this study, practical identifiability was initially analysed with the profile likelihood approach. The method determines confidence intervals where model parameters are identifiable (Lam et al. 2022; Raue et al. 2009). For *i* = 1, 2 ….*M*, where *M* is the number of parameters, profile curves are developed by fixing a single parameter, *θ*_*k,*_ over a range of 0.2–5 times its identified value in steps of 0.01 (Appendix A), while fitting the other *M*−1 parameters to the measured data (Raue et al. 2009). The weighted sum of squared residuals (${{\psi} }_{k}^{2}$) for each parameter (*θ*_*k*_)_,_ is then used as an equivalent likelihood under the assumption of zero-mean Gaussian noise as per Raue et al. (Lam et al. 2024), and is defined as

5 $$\begin{array}{c}{\psi}_{{k}}^{2}\left({\theta }_{{k}}\right)= \sum_{{i}=1}^{{N}}{\left(\frac{{{p}}_{{{aw}}_{{S}}}\left({\theta }_{{k}},{ t}{}_{{i}}, \dot{{{V}}_{{i }}} \right)-{{{p}}_{{aw}}}_{{D},{i}}}{{\upsigma }_{{D}}}\right)}^{2} ,\end{array}$$

where *N* is the number of data points; ${{{p}}_{{aw}}}_{{S}}\left({\theta }_{k}, t{}_{i}, \dot{{V}_{i}}\right)$ and ${{{p}}_{{aw}}}_{{D},{i}}$ are the simulated and measured data at time ${t_i}$, respectively; ***θ*** is the parameter vector; $\dot{{V}_i}$ is the flowrate at time ${t_i}$, and ${ \upsigma_D}$ is the measurement noise standard deviation. *σ*_*D*_ was selected as the standard deviation of the residual distribution for each breath. The value of *σ*_*D*_ was determined by analysing the residuals between the modelled and simulated breath. Confidence intervals were determined using thresholds from the Chi-Squared distribution, with α = 0.95 and one degree of freedom (DOF) for pointwise confidence intervals (Raue et al. 2009; Schwarz 2024).

The practical identifiability analysis of an in silico breath was performed on a range of EIP reductions to determine the loss of identifiability as data were truncated. Then, the same process was applied to real patient data.

### Hamiltonian Monte Carlo Simulations

RStan, an R interface for the Stan software and coding language, was used to conduct HMC simulations (Stan Development Team 2024). The main model, Eqs. (1) and (2), were rearranged to link measured airway pressure to volume and flow data (Eq. 6):

1a $${\dot{p}}_{c1}=\frac{1}{{C}_{1}}\dot{V},$$

1b $${\dot{p}}_{c2}=-\frac{1}{{R}_{2}{C}_{2}}{P}_{c2}+\frac{1}{{C}_{2}}\dot{V,}$$

2a $${p}_{c2}= {p}_{aw} -{p}_{c1}-{R}_{1}\dot{V},$$

2b $${\dot{p}}_{aw}= {\dot{p}}_{c1}+ {\dot{p}}_{c2}+{R}_{1}\ddot{V},$$

6 $${\dot{p}}_{aw} = -\frac{{p}_{aw}}{{R}_{2}{C}_{2}}+ {R}_{1}\ddot{V}+\left(\frac{1}{{C}_{1}} +\frac{1}{{C}_{2}} +\frac{{R}_{1}}{{C}_{2}{R}_{2}}\right)\dot{V } + \frac{1}{{{C}_{1}{C}_{2}R}_{2}}V.$$

Equation (6) was passed into the RStan ODE45 solver to determine *p*_*aw*_ simulations given certain values of $\varvec{\theta} .$

HMC requires random sampling to produce parameter distributions. This uses an ODE solver on Eq. 6, which requires inputs of $V, \dot{V},$ and $\ddot{V}$ which are available as part of the mechanical ventilation data ($(V, \dot{V)}$, or can be derived from the data in the case of $\ddot{V}$. To reduce computational costs in the HMC approach, a non-Bayesian method (MATLAB's *curvefit* function) was used to estimate the variables of $V, \dot{V},\text{ and } \ddot{V}$ and their parameters a-f (Eqs. 7–9). In this Bayesian framework, these variables are regarded as observables, whereas *C*_1_, *C*_2_, *R*_1_, *R*_2_ are latent model parameters that are inferred using HMC sampling in Stan.

7 $$\begin{array}{r}V= \left\{\begin{array}{r}at, \left(t<b\right)\\ c\left(t-b\right)+ab, \left(t\ge b\right)\end{array}\right.,\end{array}$$

8 $$\begin{array}{c}\dot{V}= d \text{tanh}\left(e\left(t-f\right)\right)-d\text{tanh}\left(e\left(t-g\right)\right), \end{array}$$

9 $$\begin{array}{c}\ddot{V} = de\left(\text{tanh}{\left(e\left(t - g\right)\right)}^{2} - \text{tanh}{\left(e\left(t-f\right)\right)}^{2}\right) .\end{array}$$

The fitting allowed for $V, \dot{V}, \ddot{V}$ data to be passed into the ODE45 solver as continuous functions. Fitted flow, $\dot{V},$ was subsequently used for LSQNL parameter estimation in MATLAB for comparability between HMC and PL results. Pressure, time, and volume coefficients, *a-f,* were provided as inputs to RStan. Five seconds of simulated breath data with ***θ*** = (0.015, 30, 0.01, 200) were passed into RStan to check whether the HMC’s maximum a posteriori (MAP) matched the input parameters to the model. This step did not contribute to the conclusions of the paper but was used to verify that the HMC was returning the expected parameters for an identifiable breath.

Following this validation step, the pressure and time data from a patient breath was passed into the RStan ODE solver. To manipulate the EIP duration, the data from two or four seconds before the end of inspiration was excluded from the analyses. The priors for the HMC were set based on the range of parameters identified in the cohort parameter identification, and physiological limits found by Schranz et al. (Schranz et al. 2012) (Appendix B). It was found that for a set of plausible priors informed by the frequentist results obtained with the methods described in 2.3, the priors had minimal effect on the inferred posteriors. This was confirmed by changing the prior and observing immaterial changes to parameter distributions and their MAP values.

The HMC analysis yielded posterior distributions for each parameter and an estimate of standard deviation for the model data. Bayesian methods such as HMC characterize identifiability via the convergence of chains, and the shape and modality of the resulting posterior distributions (Buckley et al. 2007). For this study, the MAPs and standard deviations of the posterior distributions were taken as the estimates for parameters and their variability for different EIP durations. Hence, identifiability can be graphically evaluated via RStan diagnostic statistics.

All analyses were undertaken on a laptop with an Intel core i7-1360P (@ 2.20 GHz) with 16 GB RAM, using MATLAB (Version R2024a 64-bit) and RStudio with RStan (Version 4.4.1 (2024-06-14 ucrt)—"Race for Your Life"). Code can be found in Appendix C.

## Results

### Patient Parameter Estimation

**Fig. 3:**
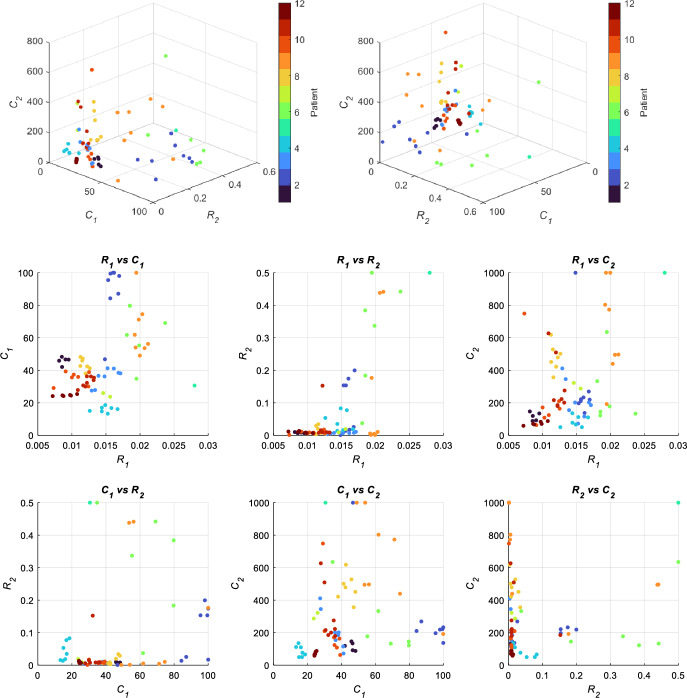
Inferred parameter values that characterise single breath dynamics for different patients are mapped in a three-dimensional space spanned by three parameters of interest: *C*_1_, *C*_2_ and *R*_2_. Shown in the top panel are two three-dimensional views of the same plot to better capture how parameters vary across the space. For some patients, the parameter values associated with single breaths are grouped in parameter space. Parameter values were estimated using LSQNL. On the bottom, two dimensional views of all parameter combinations are shown for the 73 extended EIP breaths estimated using LSQNL. Data from each patient is grouped by colour

Figure 3 shows the parameter values for the cohort of patient data that were determined using MATLAB’s *lsqnonlin* function. This corresponds to the 73 patient breaths found to have EIPs greater than 4 s (Sect. 2.3). Each colour corresponds to a unique patient. As shown, for some patients, multiple breaths fit the 4s EIP duration selection criteria.

For the same patient, similar parameter values for different breaths can be visually observed to be in proximity of one another. Means of cohort parameters showed agreement with the literature values (Schranz et al. 2012), with large standard deviations. There were outliers for static and viscoelastic compliance (*C*_1_ and *C*_2_) where parameters approached the upper bounds of identification of 100 mL/cmH_2_O and 1000 mL/cmH_2_O respectively. These bounds represent non-physiological parameters well beyond the range of values obtained by Schranz et al. 2012 (Schranz et al. 2012). These boundary values act to indicate the estimated parameters are physiologically implausible, and that results reaching these bounds suggest large parameter uncertainty.

**Fig. 4:**
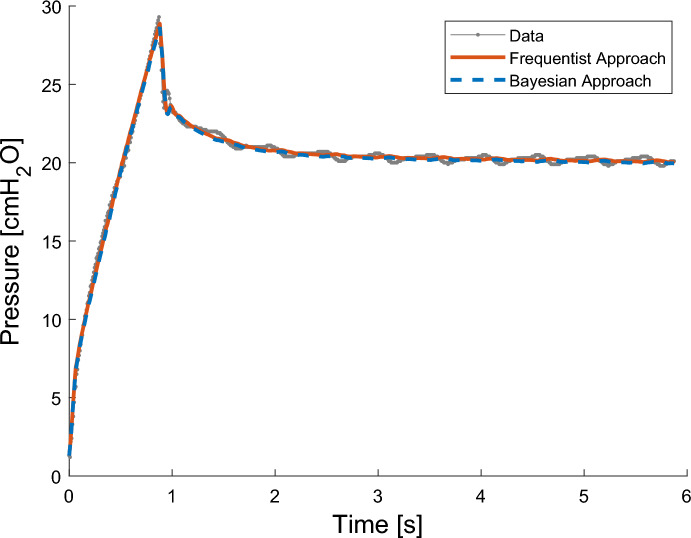
Data from one patient breath overlaid with simulation results from the frequentist approach (red) and the Bayesian approach (blue), forward simulated using Eqs. 1 and 2. In the frequentist model, parameters are estimated through maximum likelihood estimates using MATLAB’s *lsqnonlin* function. In the Bayesian model, parameters are estimated using Stan’s HMC algorithm, and MAP parameter values are used in the plot

Figure 4 shows the LSQNL and HMC fits for a highly identifiable extended EIP breath. The parameter values were taken from the MAP of the parameter distributions found by the HMC analysis. Table 1 summarises identification results.

**Table 1:** Parameter estimation results from MAP HMC values compared to LSQNL values. Results correspond to the parameter estimates used in Fig. 4

Parameter	MAP HMC value	LSQNL value
*R*_1_ [cmH_2_O s/mL]	9.58 × 10^–3^	9.86 × 10^–3^
*C*_1_ [mL/cmH_2_O]	24.5	24.4
*R*_2_ [cmH_2_O s/mL]	6.73 × 10^–3^	6.73 × 10^–3^
*C*_2_ [mL/cmH_2_O]	68.5	69.9
*σ* [cmH_2_O]	0.28	0.31

There is close agreement of parameter values between the HMC MAP and LSQNL parameter point estimates, and both fits matched the data well. Sampling took 327.8 s for the HMC. The oscillation seen in the EIP of the patient data is the result of the heartbeat, which is unmodelled in the VEM.

### Practical Identifiability

#### Practical Identifiability Given Simulated Data

**Fig. 5:**
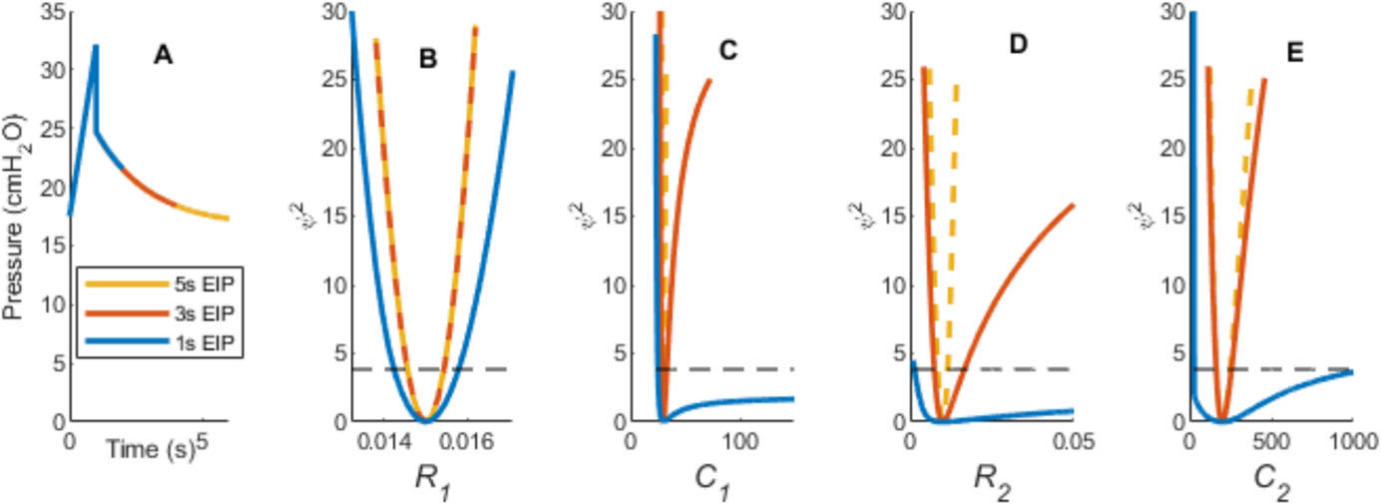
From left to right, **a** The simulated breath, with reductions of 5, 3, and 1 s to the end inspiratory pause. Figures **b** to **e** sequentially show profile likelihood curves for VEM parameters for all three EIP cases. Increase in parameter uncertainty and subsequent loss of identifiability with reduction in end inspiratory pause for forward simulated breath can be observed by the decrease in curve gradients as EIP decreases. The dotted line represents the pointwise confidence interval for 95% confidence identifiability. If the profile likelihood curve does not intersect the threshold, the parameter is not practically identifiable

Figure 5 shows the effect of end inspiratory pause length on the model identifiability for pressures simulated using ***θ*** = (0.015, 30, 0.01, 200), no noise, and an initial guess of $0.8\varvec{\theta}$.

The in silico model remained identifiable across all parameters for the 3 and 5 s EIP cases in Fig. 5. For a 1 s EIP, in blue, ${C}_{1}$ and ${R}_{2}$ lose practical identifiability on their upper bounds, shown by the curves becoming shallower and not crossing the confidence threshold (Fig. 5C, D). *C*_2_ also tends towards non-identifiability, seen in Fig. 5e. The model had a minimum error *ψ*_min_ = 0 due to lack of noise in the simulated data.

#### Practical Identifiability Given Patient Data

**Fig. 6:**
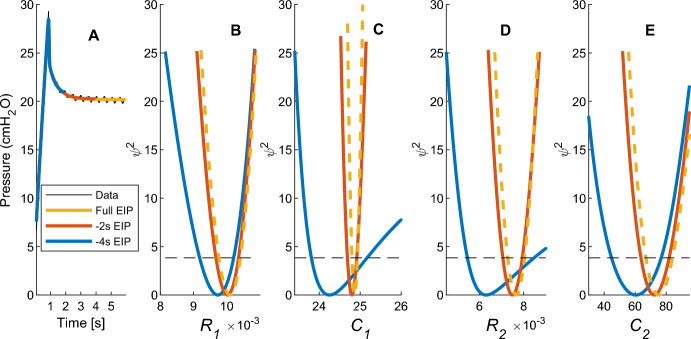
Profile likelihood curves for a patient breath with extended and reduced end-inspiratory pauses, where the dashed lines indicate 1* DOF* 95% confidence threshold. While all parameters remain identifiable for all cases of EIP for the breath, loss of identifiability can be seen in the gradient reduction with EIP reduction. Moreover, a shift in estimated parameters can be observed for the four second EIP reduction

**Fig. 7:**
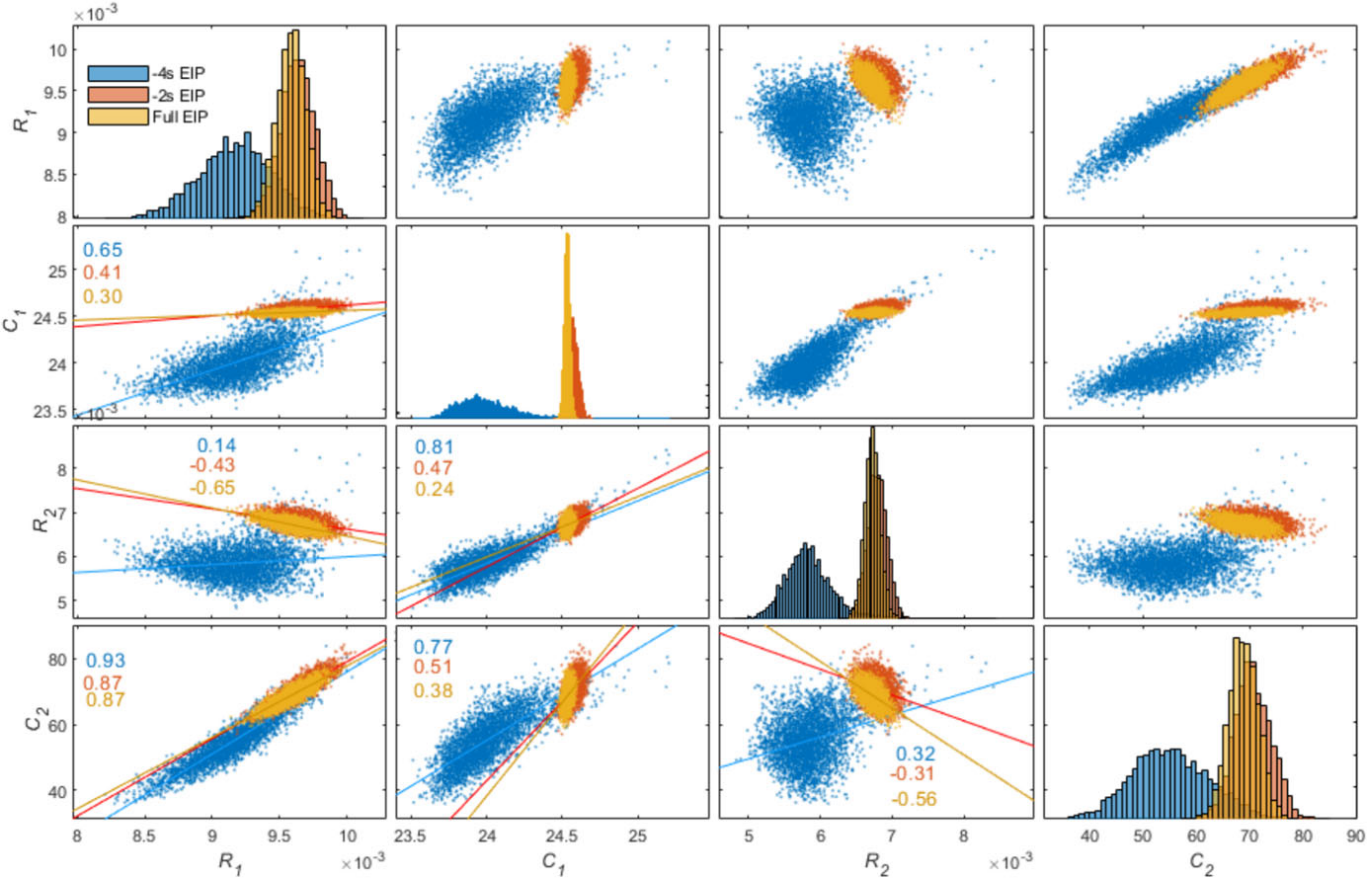
HMC posterior distributions and correlation plots for all four parameters. Correlation plots are mirrored about the diagonal and coefficients displayed on bottom section only for clarity. As shown visually, variance increases with decreasing EIP. The plots show qualitative agreement with the confidence intervals and parameter estimates from Fig. 6, particularly, the shift in distribution when there is a four second EIP reduction and the increasing variance

Identifiability analyses (PL and HMC) were performed over the identified patient breaths, and an illustrative example was selected to demonstrate the range of outcomes. A patient breath in which all four parameters were well identified was selected, and the end inspiratory pause reduced, to demonstrate loss of identifiability when the EIP is reduced in the data. Figures 6 and 7 show profile likelihood and HMC results. There was a non-zero minimum error, however this was scaled to zero for better visualization of where profile curves intersect the threshold across EIP reductions.

Table 2 summarizes the profile likelihood 95% confidence intervals, with a lower threshold of relative error of 3.8 cm H_2_O for 1 *DOF,* and HMC 95% credible intervals. Profile likelihood took 4.128 s of computational time.

**Table 2:** Profile likelihood and HMC results for a patient breath for a full EIP shown in Figs. 6 and 7. Confidence intervals (CI) and credible intervals (CrI) are shown for the frequentist and Bayesian parameters, respectively

Parameter	PL		HMC	
	Mean	95% CI	MAP	95% CrI
*R*_1_ [cmH_2_O s/mL]	9.86 × 10^–3^	[9.65 × 10^–3^, 10.07 × 10^–3^]	9.62 × 10^–3^	[9.37 × 10^–3^, 9.79 × 10^–3^]
*C*_1_ [mL/cmH_2_O]	24.43	[24.40, 24.47]	24.54	[24.49, 24.57]
*R*_2_ [cmH_2_O s/mL]	6.73 × 10^–3^	[6.49 × 10^–3^, 6.96 × 10^–3^]	6.74 × 10^–3^	[6.52 × 10^–3^, 6.94 × 10^–3^]
*C*_2_ [mL/cmH_2_O]	69.9	[61.4, 75.9]	68.0	[63.9, 74.2]

The parameters identified for both extended and reduced EIP breaths showed a good fit to the data using LSQNL for identification as part of PL analysis. For the cases with a full EIP and an EIP reduced by 2 s, the breath parameters remained highly identifiable, as seen by narrow distributions and confidence intervals in Figs. 6 and 7. However, when the EIP is reduced by 4 s, the PL curve becomes shallower and HMC distributions widen. There is also a shift in MAP and minimum error that is clearly illustrated in Figs. 6 and 7, with close graphical match between PL and HMC distributions and estimates. Table 2 shows HMC estimated narrower 95% credible intervals than the PL confidence intervals. There is a strong correlation between *C*_2_ and *R*_1_ across all lengths of EIP, however larger correlation values can be seen for the reduced EIP case.

#### Non-Identifiability with Extended EIP

**Fig. 8:**
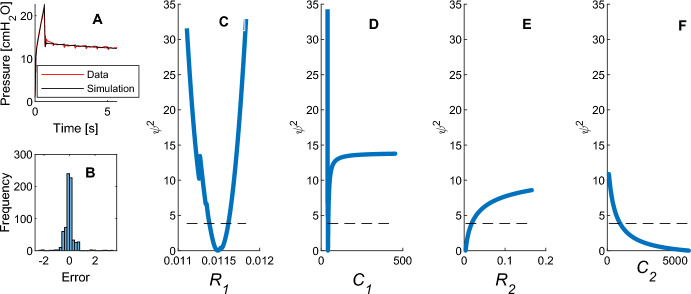
Loss of identifiability on breath with extended EIP. It can be seen in the upper left corner, Figure **a**, the model fits the data well. In the lower left corner, Figure **a**, the residual distribution is plotted. Figures **c** to **f** respectively from left to right show the profile likelihood curves for the parameters

Within the patient breaths identified for Fig. 3, there were also breaths with poor identifiability. Figure 8 shows an extended EIP breath and profile likelihood curves from a different patient. Due to the non-identifiability with an EIP duration of 5 s, 3 s and 1 s EIPs were not considered. The identified parameters were ***θ*** = (0.0121, 41.76, 0.0115, 542.0) using LSQNL.

The profile likelihood results demonstrate practical non-identifiability, particularly in the upper bounds of *R*_2_ and *C*_2_*.* Additionally, while C_1_ remains identifiable, as the value is forced toward the non-physiological domain of compliance, the *ψ*^2^ gradient diminishes. *R*_1_ remained identifiable when varied over its parameter range and the other parameters optimized. The histogram of residuals shows a normal distribution with a zero mean and a narrow distribution, with most errors between the model and data happening around the peak inspiratory part of the graph and due to the heartbeat oscillations in the data. The model fit the breath data well using the inferred parameters, except for the peak inspiratory pressure. The breath data shows a steeper pressure decay after inspiration than previous breaths.

## Discussion

The profile likelihood method is widely employed in systems biology, but its use is limited in examples within the medical field (Mitra and Hlavacek 2019). Both PL (incorporating LSQNL) and HMC estimated parameters were within close agreement. The profile likelihood analysis for the VEM showed that parameter uncertainty increased and identifiability was lost with decreasing EIP lengths for both simulated and clinical data. The changing in ${\psi }^{2}$ gradients shown in Figs. 5 and 6 indicate that all modelled parameters had increased parameter uncertainty when the EIPs were shortened. As structural identifiability of the model has been established (Schranz et al. 2012), the loss of confidence in the parameter estimates can thus be attributed to loss of information in the data and an increased influence of noise levels when EIP is shortened. *R*_1_ was shown to be identifiable across all cases (Figs. 4 and 5) while identifiability varied across other parameters. There is strong qualitative agreement between Figs. 6 and 7 comparing HMC and PL results, which aligns with previous literature comparing the methods (Simpson et al. 2020). Additionally, HMC demonstrated narrower confidence intervals as expected due to a smaller σ estimate of 0.28 compared to 0.31 for PL. The loss of identifiability observed in the data with a reduction in EIP may be due to the loss of the viscoelastic decay portion of the data, which limits the available information to which *C*_2_ and *R*_2_ can be fitted. This lack of information led to the particularly low PL gradients observed in Figs. 5 and 6.

Profile likelihood analysis was able to quantify increases in parameter uncertainty and recognise parameter non-identifiability with reduced EIP for the VEM. The HMC analysis found parameter distributions in agreement with PL analysis, and it captured parameter trade-offs during optimisation, seen in Fig. 7. These trade-offs showed that identifiability is impacted by data quantity and presence of noise (Lam et al. 2024). In these analyses, there was a clear reduction in parameter identifiability as the EIP duration was decreased. The profile likelihoods indicated when parameters were outside of the threshold for identifiability, and hence when the identified parameters were no longer practically meaningful. In biomedical modelling, it is important that the parameters correspond to the physiological condition of the patient. Therefore, it is important to perform practical identifiability analyses alongside parameter identification, especially if patient behaviour varies over time.

The pressure shown in Fig. 8A demonstrated an almost immediate relaxation phase to *P*_*plat*_ after inspiration. This may be due to the unmodelled cardiac impulses on the pressure wave. Hence, the identified value of *C*_2_ was at the upper limit that represented unphysiological values. This shows the impacts that unmodelled behaviour can have on identified model parameters. This behaviour would be well described with a first order single compartment model, which has only one compliance term. However, the two-compartment VEM is over-parameterised for this behaviour, which leads to practical identifiability issues. This shows that practical identifiability is negatively affected by a mismatch between the complexity of the model and observed behaviour (in the case of over-modelling). In biomedical modelling scenarios, inter-subject variation and disease progression both affect the presentation of patient behaviour, meaning that practical identifiability cannot be guaranteed for all patients in a population, or even for a single patient for all time. Again, this emphasises the importance of analysing practical identifiability whenever it is feasible.

To keep results interpretable, the results present an in-depth analysis of a single breath sample to directly compare PL and HMC approaches. There were also a limited number of EIPs of 3 or more seconds available in the dataset to gain a more accurate understanding of the effects of EIP duration. Ventilation settings and respiratory manoeuvres were not homogenous across the cohort, which resulted in differences in volume, flow rate, and PEEP inputs, which affected pressure profiles. Identifiability may be further affected by the model’s inability to capture effects like the heartbeat, or instantaneous relaxation (Fig. 8A). However, this further indicates trade-offs between the viscoelastic terms, and this non-identifiability would be expected due to human lung physiology and disease state variability (Dickson et al. 2014).

This paper demonstrates a comparison of PL with HMC parameter estimation methods. A similar comparison, done by Simpson et al. (Simpson et al. 2020) for cell invasion modelling, has now been applied in a respiratory modelling context. For the VEM context presented here, methods followed Simpson et al.’s recommendation to begin with PL to determine identifiability, then proceed with HMC to gain more information on parameter interactions. PL exhibited a trade-off between *C*_1_ and *R*_2_ (Figs. 5 and 6), where both parameters become less identifiable with reduced EIP, while HMC was able to clarify the nature of this relationship as shown in Fig. 7, where it showed a strong correlation between these parameters for the case when EIP is reduced. Simpson et al. also found that the HMC simulations were one order of magnitude slower than PL simulations, which agreed with this paper’s implementation, where PL and HMC took 4.128 s and 327.8 s seconds of computational time, respectively, for estimating parameters for one breath.

Plateau pressure is the pressure applied by the ventilator in the alveolae, and it can lead to ventilator induced lung injury if too high (Warner et al. 2013). It can be read from a ventilator screen after a sufficiently long EIP, or alternatively, it can be estimated by using the *C*_1_ parameter fitted from the viscoelastic model and the tidal volume. With reduced EIPs, estimation of *C*_1_ would be less robust (Figs. 5 and 6), and thus there would be lower confidence in *P*_*plat*_. Hence, incorporating practical identifiability analysis in clinical settings may allow for prediction of a confidence interval for *P*_*plat*_. This confidence interval may provide clinical decision support or show when an extended EIP is needed to more fully understand the patient state.

Quantifying identifiability can provide confidence in the identified parameters. When clinical decisions are supported by parameter identification of patient data using models, quantifying uncertainty of the results to ensure that they accurately reflect the patient condition is critical. The parameter identification values for the cohort of this study are consistent with prior studies using the same data (Schranz et al. 2012), and the practical identifiability outcomes offer insights into which parameters were well-identified, and which may be uncertain. The loss of identifiability observed in the viscoelastic terms show the VEM may not be suitable for capturing lung behaviour for ARDS patients, and a more adaptable model may be required. While clinicians currently have protocols for identification of patient parameters, the addition of parameter identifiability information could improve risk assessment for treatments and/or ventilation settings.

## Conclusion and Outlook

This study provides a new application of profile likelihoods and HMC to lung modelling, with potential to be used in a clinical setting to help inform decision making. Parameter identifiability was analysed for a range of end-inspiratory-pause durations, both in silico and patient data. The identifiability analysis was able to determine parameters with poor identifiability (via PL) and correlations between model parameters (via HMC). The PL and HMC results for the VEM corroborate previous literature comparing the two methods, showing strong agreement between these methods for practical identifiability analysis. This study found that for the VEM, reduction in breath EIP during mechanical ventilation leads to an increase in parameter uncertainty that varies between breaths and patients. HMC, although more computationally expensive, was found to support PL results and offer further insights into parameter behaviour, particularly which parameters exhibit trade-off behaviour. Static resistance (*R*_1_) has low parameter uncertainty in all cases, with large variations in how well *C*_1_, *R*_2_, and *C*_2_ are identified for different breath conditions. Trade-offs between *R*_2_ and *C*_1_ are shown by the correlation between these parameters in the HMC results and the loss of their identifiability with reduced EIP. Identifiability of *C*_1_ varied across breaths and patients, inhibiting its use for *P*_*plat*_ prediction.

This research focused on describing the practical identifiability of the VEM fitted to patient data. Future work could include linking identifiability to patient condition and disease state, as increased stiffness in the lungs could cause a reduction in second order behaviour and thus limit identifiability. This could additionally be extended by employing Bayesian estimation of viscoelastic parameters to improve robustness of *C*_1_ prediction for plateau pressure estimation without the need for an EIP. However, successfully implementing this modelling would require validation on more patient data across a wide range of relevant patient responses to treatment. These methods have potential to improve confidence in identification of model parameters and allow models to be further employed in clinical settings to aid decision-making in the ICU.

## Appendices

### A. Parameter ID and Profile Likelihood Boundaries


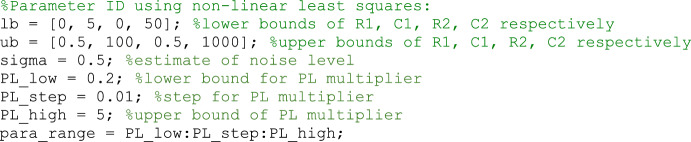


Xxx

### B. HMC Settings

Sampling was set to seed = 123, using 4 chains, 1000 warm up iterations and 2000 iterations total. Sampler algorithm settings of stepsize = 0.01, adapt_delta = 0.99.

Priors used in the model were:

R1 ~ N(0.01, 0.1);

C1 ~ N(30, 20);

R2 ~ N(0.05, 0.1);

C2 ~ N(200, 150).

where N($\mu , \sigma )$, refers to the normal distribution with mean $\mu$ and standard deviation $\sigma$.

### C. Stan Code

The Stan code below was implemented to infer probabilistic VEM parameters.
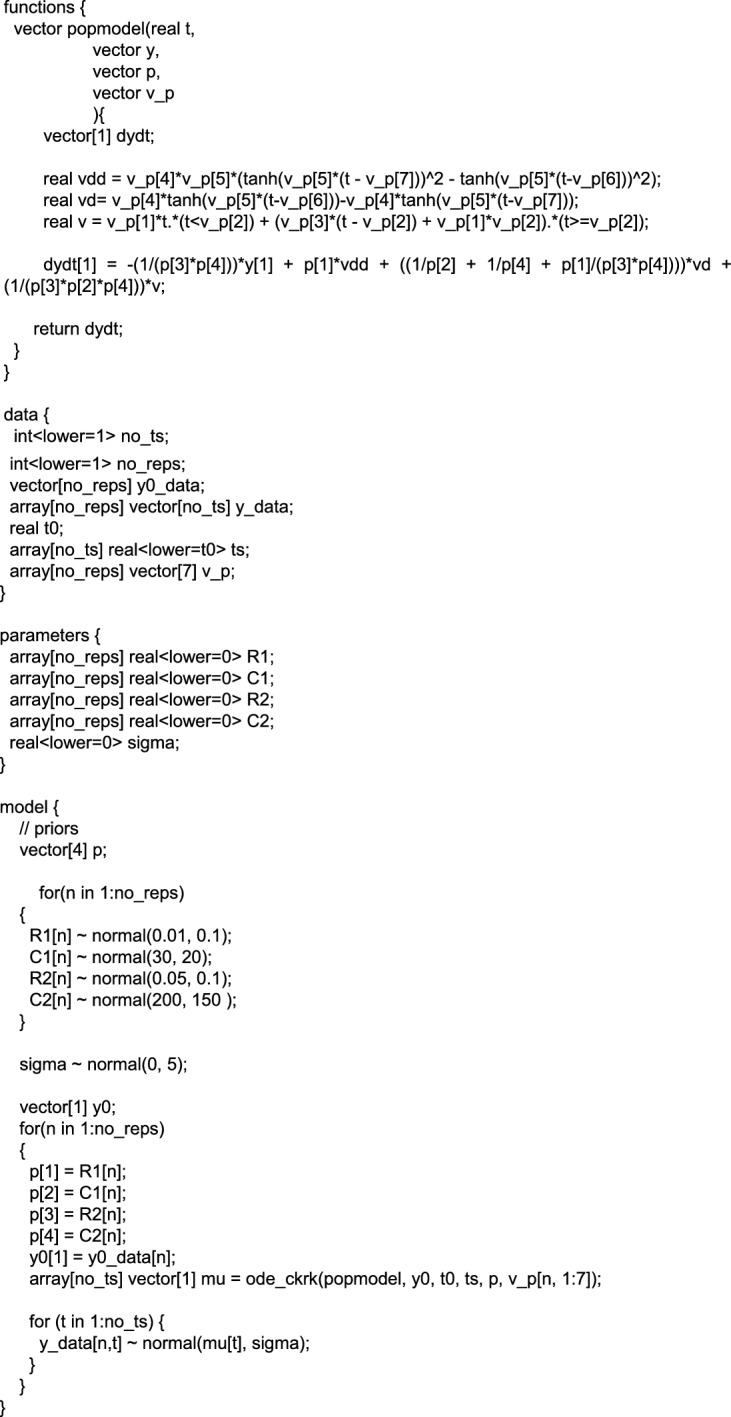


## Data Availability

Historical data agreements preclude the availability of data from the ARDS study.
